# Innovative Nanomedicine Delivery: Targeting Tumor Microenvironment to Defeat Drug Resistance

**DOI:** 10.3390/pharmaceutics16121549

**Published:** 2024-12-03

**Authors:** Wenjun Meng, Li Huang, Jiamin Guo, Qing Xin, Jiyan Liu, Yuzhu Hu

**Affiliations:** 1 Department of Radiation Oncology, Cancer Center, West China Hospital, Sichuan University, Chengdu 610041, China; 2 Department of Biotherapy, Cancer Center, West China Hospital, Sichuan University, Chengdu 610041, Chinaliujiyan1972@163.com (J.L.); 3 Division of Abdominal Tumor Multimodality Treatment, Cancer Center, West China Hospital, Sichuan University, Chengdu 610041, China

**Keywords:** nanomedicine delivery, tumor microenvironment, drug resistance

## Abstract

Nanodrug delivery systems have revolutionized tumor therapy like never before. By overcoming the complexity of the tumor microenvironment (TME) and bypassing drug resistance mechanisms, nanotechnology has shown great potential to improve drug efficacy and reduce toxic side effects. This review examines the impact of the TME on drug resistance and recent advances in nanomedicine delivery systems to overcome this challenge. Characteristics of the TME such as hypoxia, acidity, and high interstitial pressure significantly reduce the effectiveness of chemotherapy and radiotherapy, leading to increased drug resistance in tumor cells. Then, this review summarizes innovative nanocarrier designs for these microenvironmental features, including hypoxia-sensitive nanoparticles, pH-responsive carriers, and multifunctional nanosystems that enable targeted drug release and improved drug penetration and accumulation in tumors. By combining nanotechnology with therapeutic strategies, this review offers a novel perspective by focusing on the innovative design of nanocarriers that interact with the TME, a dimension often overlooked in similar reviews. We highlight the dual role of these nanocarriers in therapeutic delivery and TME modulation, emphasize their potential to overcome drug resistance, and look at future research directions.

## 1. Introduction

Cancer is one of the major diseases leading to high mortality and morbidity worldwide, and its complex tumor microenvironment (TME) and highly heterogeneous nature pose great challenges for treatment [1,2]. The TME not only provides physical support for tumor cell growth, invasion, and metastasis, but also largely influences the therapeutic efficacy and drug tolerance [3]. The TME consists of a wide range of cellular and noncellular components, including mesenchymal cells, immune cells, extracellular matrix (ECM), blood vessels, and dissolved growth factors and cytokines. Together, these components form a complex biochemical and physical barrier, limiting the efficiency of conventional drugs to enter the tumor [4,5]. The TME has emerged as a crucial determinant of cancer progression, metastasis, and resistance to multiple therapies.

With the development of biomedical technology, nanotechnology provides revolutionary new ideas for drug delivery [6]. Nanomedicine, as a multidisciplinary research field, aims to achieve efficient drug delivery, precise targeting, and reduction of systemic toxicity by designing micro- and nano-scale materials and carriers [7]. Especially in tumor therapy, nanocarriers are not only able to passively aggregate at the tumor site through enhanced permeability and retention (EPR) effects, but are also able to actively target specific regions of the TME, such as hypoxic and acidic zones, through surface modification and functionalized design, thus improving therapeutic selectivity and effectiveness [8]. For instance, the acidic pH of the TME has inspired pH-responsive nanoparticles that release therapeutic agents specifically in the tumor site. A notable example is a pH-sensitive polymer–lipid hybrid nanocarrier that ensures precise drug release, minimizing systemic toxicity [9]. Moreover, hypoxia-targeting nanotechnologies have also gained momentum. Oxygen-releasing nanoparticles or hypoxia-sensitive nanocarriers have been designed to enhance the delivery of oxygen-dependent drugs and alleviate hypoxia-induced resistance [10]. These systems leverage the overexpression of hypoxia-inducible factors (HIFs) in the TME to enhance therapeutic precision.

However, despite the fact that targeted delivery to the TME shows great potential, tumor drug resistance remains a major challenge in current tumor therapy [11]. Drug resistance includes not only innate resistance, but also acquired resistance acquired by tumor cells during treatment through mechanisms such as gene mutations and epigenetic changes [12]. Certain features in the TME, such as high mesenchymal pressure and the overexpression of drug efflux proteins, can also further exacerbate the drug resistance problem [13]. In response to these challenges, nanotechnology has demonstrated unique advantages in overcoming tumor drug resistance through the design of smart carriers, such as pH-responsive carriers, oxygen-sensitive carriers, and heat- or light-sensitive carriers [14].

In this context, this review will continue to systematically review and summarize the current applications of nanotechnology in drug delivery systems, especially those nanocarriers that can target the TME. Unlike previous reviews, we aim to highlight cutting-edge innovations in smart nanocarrier design and their dual roles in therapeutic delivery and TME modulation. Moreover, while numerous reviews have explored the role of nanomedicine in targeting the TME, this review seeks to provide a fresh perspective by focusing on the integration of advanced design strategies in nanocarrier systems and their application in overcoming drug utility and tumor drug resistance. Despite significant progress, several challenges persist in nanomedicine, including scaling up production, ensuring batch consistency, addressing long-term safety concerns, and facing artificial intelligence (AI) trends. Therefore, this paper will also discuss the biocompatibility and safety of nanodrug delivery systems and the opportunities and challenges they face during clinical translation.

## 2. Complexity of the Tumor Microenvironment

The TME is a complex ecosystem consisting of tumor cells together with their surrounding non-tumorigenic components [5]. It not only provides the necessary support for tumor cell growth, invasion, and metastasis, but also influences tumor drug resistance, immune escape, and therapeutic response through a variety of mechanisms. The unique characteristics of the TME, such as hypoxia, an acidic environment, and high mesenchymal pressure, often constitute a physical barrier to conventional drug delivery and therapy [15]. Understanding the complexity of TME is pivotal to designing efficient targeted drug delivery systems [16].

### 2.1. Composition of the Tumor Microenvironment

**Figure 1:**
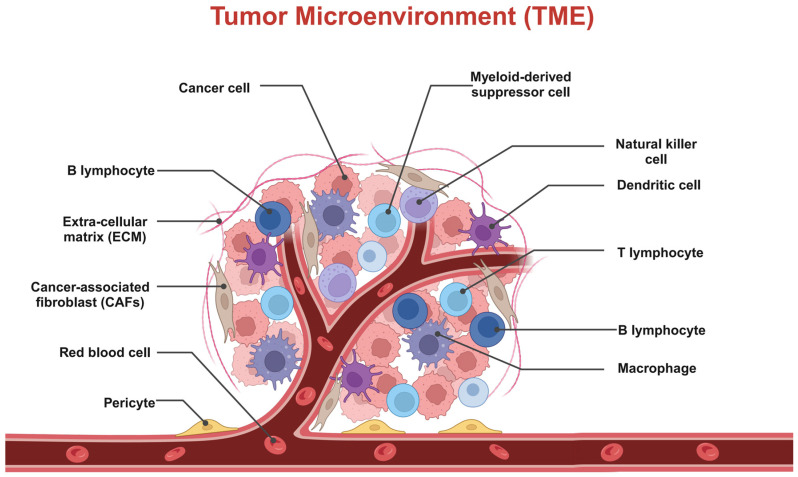
Composition of the TME. The TME consists of a variety of cellular and non-cellular components, including tumor-associated fibroblasts (CAFs); immune cells such as macrophages, T cells, natural killer cells, and endothelial cells; pericytes; the extracellular matrix (ECM); and growth factors and cytokines. Created with BioRender.com.

TME consists of a variety of cellular and non-cellular components, including tumor-associated fibroblasts (CAFs), immune cells (e.g., macrophages, T cells, and natural killer cells), endothelial cells, pericytes, and the ECM, as well as growth factors and cytokines, among others (Figure 1) [17]. The interactions between these components regulate tumor progression and treatment tolerance [18]. For example, CAFs contribute to the acquisition of invasiveness and drug resistance by tumor cells through the secretion of cytokines and ECM components [19], whereas immunosuppressive cells such as tumor-associated macrophages (TAMs) reduce the effectiveness of antitumor immunity through immune escape mechanisms [20]. Non-cellular components, especially the ECM, constitute a physical barrier to the TME [21]. The ECM consists of a variety of macromolecules, such as collagen, elastin, and glycoproteins, which form a high-density mesh structure [22]. This structure not only restricts drug penetration, but also generates high interstitial pressure within the tumor, further hindering drug delivery [23].

### 2.2. Hypoxic Zones

Hypoxia is one of the typical features of the TME, mainly due to fast-growing tumor cells exceeding the ability of neovascularization to supply oxygen [24]. Tumor cells exhibit a series of adaptive responses in a hypoxic environment, such as the up-regulation of HIFs, enhanced angiogenesis, and glycolytic metabolism [25]. These changes not only promote tumor growth and metastasis, but are also closely related to the tolerance of chemotherapy and radiotherapy [26]. Many chemotherapeutic drugs, such as doxorubicin and cisplatin, rely on oxygen to produce reactive oxygen species (ROS) to kill tumor cells, but the production of ROS is significantly reduced under hypoxic conditions, which decreases the killing effect of the drugs [27,28]. To address these challenges, nanotechnology provides a solution by developing hypoxia-sensitive carriers. For example, some nanoparticles are designed to release drugs in hypoxic environments, or oxygen-carrying nanomaterials (e.g., superparamagnetic iron oxide nanoparticles) are used to increase the oxygen content at the tumor site, thereby enhancing drug efficacy [29].

### 2.3. Acidic Environments

Rapid tumor cell growth contributes to the formation of its local acidic microenvironment [30]. This acidity is due to the massive reliance of tumor cells on anaerobic glycolysis, leading to the excessive accumulation of lactic acid [31]. The acidic character of the TME not only promotes tumor aggressiveness, but also enhances the tolerance of tumor cells to a wide range of chemotherapeutic agents [32], as many drugs are less active under acidic conditions or have difficulty penetrating the cell membrane due to low extracellular pH [33]. In response to this problem, pH-responsive nanocarriers have been developed [34]. These carriers can trigger drug release in the acidic environment of tumors or enhance drug accumulation at the tumor site by changing their surface charge and physical properties under acidic conditions [35]. For example, some nanoparticles rapidly release encapsulated anticancer drugs by undergoing dissolution or deformation in acidic environments, thereby enhancing their therapeutic effects [36].

### 2.4. High Interstitial Pressure

Another remarkable feature of the TME is its high interstitial pressure [37]. The overproliferation and accumulation of tumor cells and their surrounding ECM lead to abnormal physical pressure inside the tumor [38]. This pressure not only inhibits the normal function of the tumor vasculature, but also hinders drug penetration through blood vessels to the core of the tumor [39]. High interstitial pressure also further exacerbates drug diffusion obstacles within the tumor tissue by increasing the density and rigidity of the ECM [23]. Nanodrug delivery systems can overcome this obstacle by modulating their physical properties, such as particle size, shape, and surface modifications [40]. For example, some finely designed nanoparticles can increase drug distribution inside the tumor by altering their own structure to reduce the obstruction of high interstitial pressure when penetrating the ECM [41]. In addition, nanocarriers can reduce ECM density by carrying enzymes capable of degrading ECM components, thereby improving drug diffusion and delivery [42].

The complexity of the TME is an important challenge in tumor therapy, and various factors including hypoxia, an acidic environment, and high interstitial pressure limit the delivery and efficacy of conventional drugs [43]. Nanodrug delivery systems offer new hope for overcoming these barriers by targeting these features of the TME [44].

## 3. Nanotechnology Targeting in the Tumor Microenvironment

**Figure 2:**
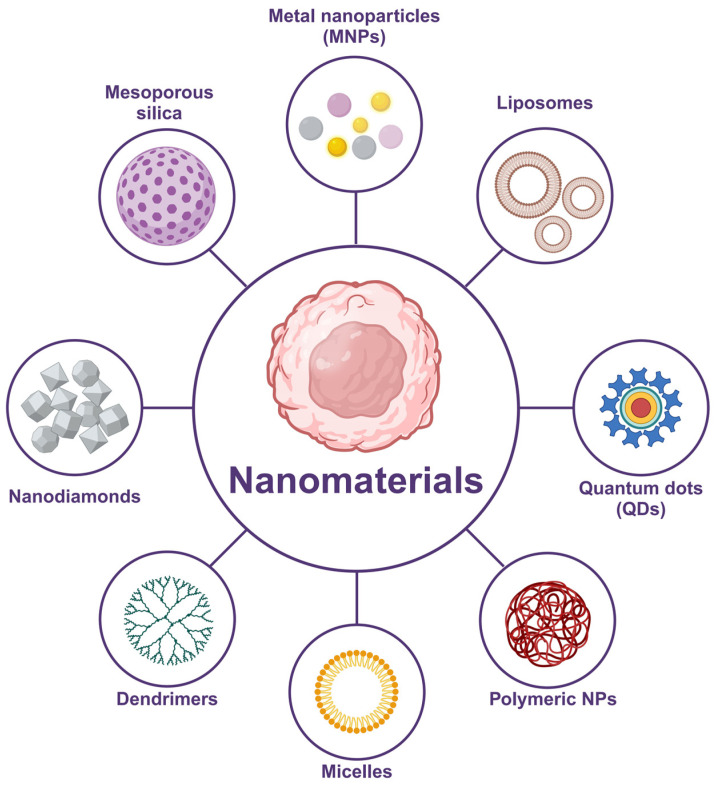
Schematic diagram of nanomaterials employed to diagnose and treat cancers, including nanodiamonds, mesoporous silica, metal nanoparticles (MNPs), liposomes, quantum dots (QDs), polymeric NPs, micelles, and dendrimers. Created with BioRender.com.

The application of nanotechnology in tumor therapy has shown great potential, especially in the design of nanocarriers targeting the TME [45]. Conventional chemotherapeutic drugs usually face the problems of drugs not easily reaching the core tumor region, non-specific distribution of drugs in normal tissues, and drug resistance caused by the TME [46]. Various nanocarriers can enhance drug delivery efficiency and reduce damage to normal tissues by designing them to target the unique characteristics of the TME, such as hypoxia, acidity, and high interstitial pressure (Figure 2) [47]. In the following section, we will discuss in detail how nanocarriers can target different features of TME to achieve precise drug delivery and effective treatment.

### 3.1. Targeted Delivery of Nanocarriers in the Hypoxic Zone

**Figure 3:**
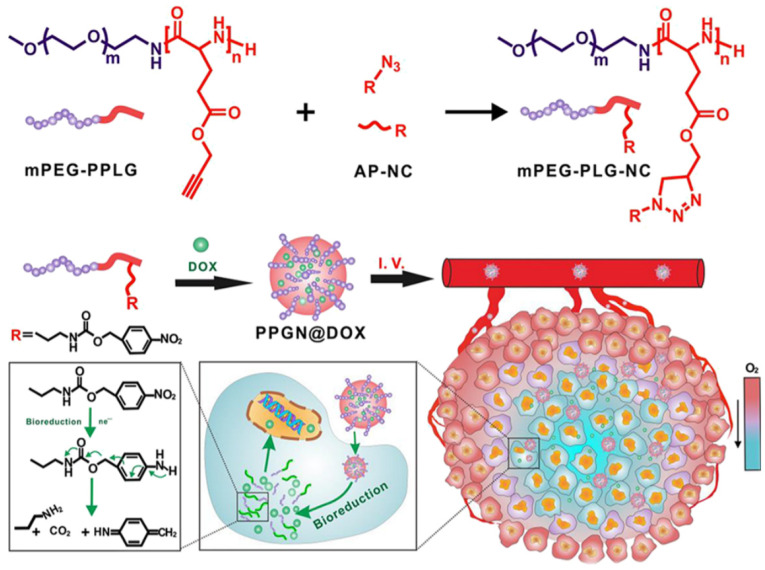
The p-nitrobenzyl groups in mPEG-PLG-NC are supposed to be reduced to p-aminobenzyl groups by overexpressed nitroreductase in hypoxic cells and undergo spontaneous fragmentation via 1,6-elimination, resulting in hypoxiaresponsive drug release. (Reprinted with permission from [58]. Copyright 2020 American Chemical Society).

As one of the typical features of the TME, hypoxia usually results in the formation of hypoxic zones within the tumor due to rapid tumor growth exceeding the oxygen supply capacity of blood vessels [48]. This hypoxic environment not only limits the growth of tumor cells, but also promotes the invasive and metastatic potential of the tumor and resistance to radiotherapy and chemotherapy [49]. Radiotherapy relies on oxygen to generate free radicals in order to kill cancer cells, and hypoxic tumor cells show higher tolerance to radiotherapy [50]. Also, many chemotherapeutic agents, such as doxorubicin and cisplatin, have decreased activity under hypoxic conditions [51]. Therefore, targeting drug delivery to the hypoxic region of the tumor is an important strategy to improve the efficacy of tumor therapy [52,53]. The binding of paclitaxel to the quinone moiety results in the formation of a prodrug that can be reduced by reductase in an oxygen-deprived environment [54]. This process releases paclitaxel, which then undergoes selective activation of the drug. This strategy effectively mitigates the toxic effects on normal tissues while enhancing the precision and efficacy of tumor therapy [55]. Nanotechnology provides an effective means to overcome the hindrance of hypoxia on tumor therapy [56]. Hypoxia-sensitive nanocarriers take advantage of the difference in oxygen concentration in the TME to perform specific drug release or enhance therapeutic effects by sensing low oxygen levels [57]. For example, some nanoparticles are designed to break down or release active drugs under hypoxic conditions, ensuring effective drug delivery within the hypoxic zone of the tumor (Figure 3) [58]. Researchers have designed prodrugs that covalently link antitumor drugs to hypoxia-responsive groups, including nitro, quinone, and azo [59]. As an illustration, common antitumor drugs such as doxorubicin, cisplatin, and paclitaxel can be released by forming covalent prodrugs with nitro and quinone groups, etc. In hypoxic environments, the responsive groups are reduced by biological reductase, thereby releasing the active drug. For example, doxorubicin, after forming a covalent bond with a nitro group, is capable of releasing adriamycin in hypoxic tumor tissues through the action of a biological reductase, which directly attacks tumor cells [60]. Superparamagnetic iron oxide nanoparticles can carry oxygen and release it upon reaching the hypoxic region to improve the local oxygenation status, thus enhancing the radiation therapy effect [29]. In addition, superparamagnetic iron oxide nanoparticles can be used as drug carriers to deliver chemotherapeutic drugs precisely to the hypoxic region [29].

In a novel approach, superparamagnetic iron oxide nanoparticles have been utilized for dual purposes: oxygen delivery and targeted drug release. These nanoparticles improve oxygenation within the tumor and release cisplatin specifically in hypoxic regions, significantly enhancing its anticancer efficacy. For example, magnetic iron oxide nanoparticles containing cisplatin are able to release the drug in the hypoxic region, thus effectively increasing the anticancer activity of the drug [61]. Reduction-sensitive nanoparticles release drugs by reacting with glutathione (GSH) in a reducing environment [62]. Tumor cells, especially in hypoxic regions, usually have high levels of reducing molecules such as GSH [63], and these nanoparticles are able to degrade rapidly upon encountering GSH, thereby releasing the drug and enhancing tumor killing [64]. Another type of strategy is to increase the local oxygen supply to the tumor directly through nanocarriers, thereby increasing the sensitivity of the tumor to radiotherapy and chemotherapy [65]. For example, nanocarriers carrying catalase can catalyze the decomposition of hydrogen peroxide to produce oxygen, alleviate the hypoxic state of tumors, and enhance the effect of radiotherapy [66,67]. This strategy enhances the effect of chemotherapeutic agents while increasing the sensitivity of radiotherapy.

### 3.2. Smart Nanocarriers for Acidic Microenvironments

The acidity of the TME is caused by the abnormal metabolism of cancer cells, especially the accumulation of large amounts of lactic acid during anaerobic glycolysis, which leads to a significantly lower local pH in tumors than in normal tissues [68]. The acidic TME not only affects the behavior of tumor cells, but also adversely affects the activity of many chemotherapeutic drugs [32]. Conventional chemotherapeutic agents may lose their chemical stability in an acidic environment or have difficulty in effectively penetrating the tumor cell membrane, thus reducing the therapeutic efficacy [69]. To cope with the acidic environment of tumors, pH-responsive nanocarriers have been widely studied [34]. These nanocarriers are able to undergo structural changes or decomposition in acidic environments, thus releasing drugs or exposing functional molecules for drug-specific release [36].

**Figure 4:**
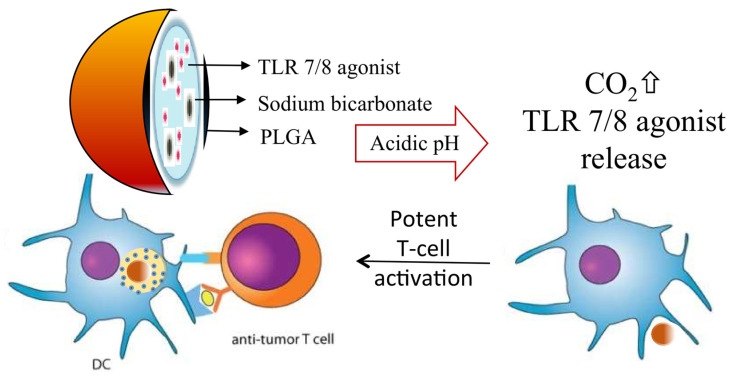
Acidic pH-responsive poly(lactide-co-glycolide) (PLGA) nanoparticles for the endo-lysosome-specific release of 522, a novel TLR7/8 agonist. Bicarbonate salt was incorporated into the new formulation to generate carbon dioxide (CO_2_) gas at acidic pH, which can disrupt the polymer shell to rapidly release the payload, and the polymer shell can be used for the release of the payload. (Reprinted with permission from [71]. Copyright 2018 Royal Society of Chemistry).

The following are a few common designs: pH-sensitive polymer nanoparticles are usually composed of pH-sensitive polymers, such as PLGA or poly(ethylene glycol) (PEG)-modified polymers [70]. These polymers are able to rapidly depolymerize in acidic environments, releasing the encapsulated chemotherapeutic drug (Figure 4) [71]. For example, pH-responsive PLGA nanoparticles are able to rapidly release drugs such as doxorubicin under the acidic conditions of the TME, increasing the concentration of drug accumulation in the tumor [72].

Liposomes are a common drug delivery system, which can be deformed or cleaved in acidic microenvironments through pH-sensitive lipid bilayer structures for rapid drug release [73]. For example, pH-responsive liposomes can release drugs in acidic regions of tumors and rapidly penetrate tumor cell membranes to improve drug delivery efficiency [74].

Certain nanoparticles are capable of self-assembling into large particles in the acidic environment of tumors, thereby prolonging their retention time and promoting sustained drug release. For example, researchers have developed a nanoparticle based on amphiphilic molecules that are able to self-assemble into large molecular structures in acidic environments, which effectively enhances the retention efficiency of drugs in tumors [75].

Hydrogel-based systems, especially pH-sensitive hydrogels, have been utilized to encapsulate and release anticancer drugs specifically in acidic TME conditions. For instance, hydrogels based on chitosan and hyaluronic acid have demonstrated pH-sensitive drug release capabilities, enabling the controlled release of doxorubicin in response to acidic conditions, which is beneficial for tumor-site-specific administration [76]. Similarly, hollow mesoporous silica nanoparticles (HMSNs) modified with hyaluronic acid have been developed as a pH-responsive and tumor-targeted drug delivery system [77]. This system allows for the encapsulation of both doxorubicin and photosensitizers, facilitating chemo-photodynamic combination therapy. The pH sensitivity of the HMSNs enables efficient drug release in slightly acidic environments, thereby improving drug availability at the tumor site. Moreover, the use of poly(tannic acid) fabricated nanocarriers has been explored for their reversibly pH-responsive properties, which allow for regulated drug release in a “release–stop–release” manner. This feature is particularly advantageous in reducing secondary side effects associated with traditional chemotherapeutics, as the nanocarriers can prevent the leakage of incompletely released drugs upon re-entering the bloodstream [78].

Metal–organic frameworks (MOFs) have recently gained attention for their unique ability to release drugs in response to pH changes [79]. This property is particularly advantageous in the field of targeted drug delivery, where the goal is to minimize side effects and enhance the efficacy of therapeutic agents. MOFs can be engineered to respond to the acidic microenvironments typical of tumor tissues, allowing for the controlled release of encapsulated drugs precisely where they are needed [80]. For instance, some MOFs have been designed with stimulus-responsive characteristics that enable them to release their payloads in response to pH variations, thereby improving the selectivity of drug delivery to cancer cells [81].

Charge-reversible nanoparticles have been developed to enhance the interaction with tumor cells under acidic conditions. In one study, these nanoparticles maintained a negative charge in physiological conditions but switched to a positive charge in the acidic tumor microenvironment, thereby facilitating enhanced internalization by tumor cells [82]. This charge reversal mechanism not only improves the targeting efficiency of the nanoparticles, but also reduces non-specific interactions that can lead to rapid clearance from the bloodstream.

### 3.3. Nanosystems for High Interstitial Pressures

In the TME, the excessive proliferation of tumor cells and their surrounding ECM leads to the formation of abnormal physical pressure inside the tumor, i.e., high interstitial pressure [38]. High interstitial pressure reduces the opportunity for drugs to enter the tumor through blood vessels by compressing blood vessels and lymphatic vessels within the tumor [83]. In addition, the high density and complex structure of the tumor ECM constitute a significant physical barrier to the diffusion of macromolecular drugs and nanoparticles, preventing drugs from effectively penetrating into the core region of the tumor [84]. To overcome the challenges posed by high interstitial pressure, researchers have developed several nanotechnology strategies.

By adjusting the size and shape of the nanocarriers, their permeability in tumor tissues can be improved [85]. For example, smaller-sized nanoparticles (20–50 nm) can penetrate the ECM more easily than larger particles and, thus, are better suited to cope with high interstitial pressure [86]. In addition, long rod-shaped or wire-shaped nanocarriers are able to penetrate the pores in the ECM more efficiently, improving the efficiency of drug delivery within the tumor [87].

To further enhance drug diffusion in the tumor, nanocarriers can carry or release enzymes that degrade the ECM, such as hyaluronidase [88]. These enzymes are able to degrade macromolecular components such as hyaluronic acid in the ECM and reduce the ECM density, thereby increasing drug penetration and diffusion. Those nanoparticles are therefore able to release hyaluronidase after reaching the TME, thus effectively degrading the ECM and enhancing drug delivery. For example, a design that combines recombinant human hyaluronidase PH20 on the surface of PLGA-PEG nanoparticles can significantly enhance its ability to function in tumors without affecting the blood circulation time of the nanocarrier [89].

Designing nanocarriers with multifunctionality is also an effective way to cope with high interstitial pressure [61]. For example, researchers have developed a multifunctional nanocarrier carrying both antitumor drugs and ECM-degrading enzymes, which, upon entering the TME, first releases the ECM-degrading enzymes to reduce interstitial pressure, and then releases the drugs to ensure that the drugs penetrate more readily and work in the tumor core region [90]. The design of nanocarriers provides innovative solutions for targeting complex features in the TME [57].

By targeting hypoxia, acidity, and high interstitial pressure, nanocarriers are able to significantly improve drug delivery efficiency and overcome the impediments of the TME to therapy. These technologies provide important tools for precision tumor therapy and new hope for overcoming the challenges of tumor treatment.

## 4. Mechanisms of Nanomedicine Delivery Systems Against Tumor Drug Resistance

Tumor drug resistance is one of the major challenges in cancer treatment, including both primary resistance and acquired resistance that develops gradually during treatment [91]. Tumor resistance may originate from a variety of mechanisms, such as the overexpression of drug efflux proteins, enhanced DNA repair capacity, escape from apoptosis, and other regulatory factors in the TME [92]. These mechanisms greatly diminish the effectiveness of conventional chemotherapy and targeted therapy. In recent years, nanotechnology has provided a series of innovative drug delivery systems, and through intelligent design, nanocarriers are not only able to overcome tumor drug resistance mechanisms, but also to reverse drug resistance and enhance the sensitivity of tumor cells to drugs [93]. Its mechanism mainly involves targeted release, inhibiting drug efflux, enhancing drug uptake by cells, reversing key signaling pathways associated with drug resistance (such as PI3K/Akt and MAPK), targeting transcription factors (such as NF-κB and STAT3), affecting the expression of drug resistance genes, activating the immune system, and promoting the apoptosis of tumor cells.

### 4.1. Nanocarrier Design to Bypass Resistance Mechanisms

**Figure 5:**
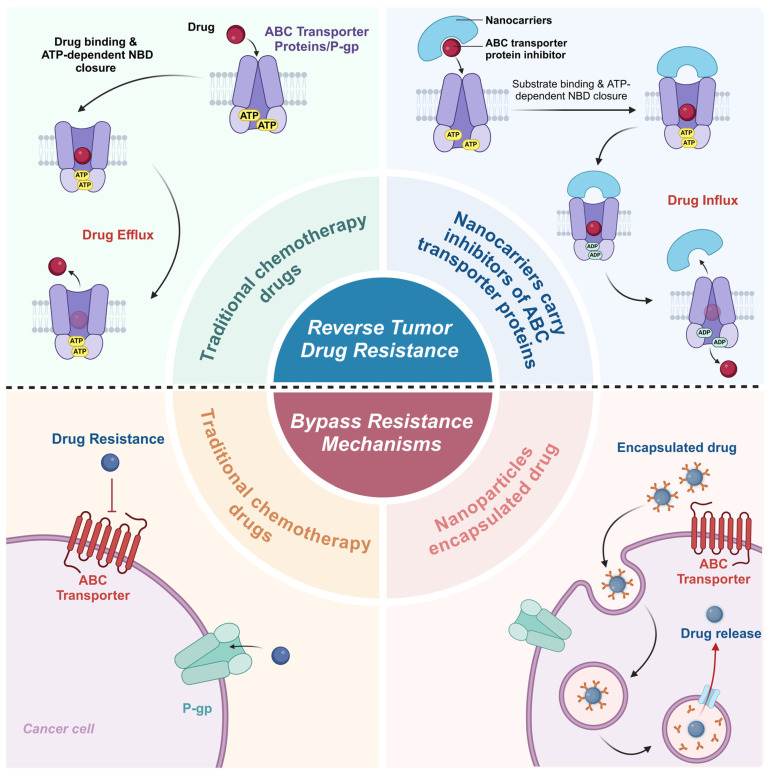
Nanocarrier strategies to overcome tumor drug resistance. Tumor drug resistance mediated by ATP-binding cassette (ABC) transporters is addressed through two strategies: reversal and bypass. Traditional chemotherapy drugs are effluxed by ABC transporters, reducing efficacy (**top left**). Reversal involves nanocarriers co-delivering drugs and transporter inhibitors to block efflux and restore drug sensitivity (**top right**). The bypass strategy uses drug-encapsulated nanoparticles to avoid transporter recognition, enabling intracellular drug release after uptake (**bottom right**), improving efficacy compared to unencapsulated drugs (**bottom left**). Created with BioRender.com.

Tumor cells develop drug resistance in various ways, among which multidrug resistance (MDR) is the most common [94]. MDR is usually triggered by the overexpression of ATP-binding cassette transporters (ABC transporters) such as P-glycoprotein (P-gp), which actively expel chemotherapeutic drugs from tumor cells, thus making the drug concentration insufficient for therapeutic effects [95]. Nanodrug delivery systems can bypass the drug efflux mechanism by encapsulating anticancer drugs in nanoparticles and avoiding their direct contact with ABC transporter proteins (Figure 5). For example, liposome and polymer nanoparticles can effectively encapsulate conventional chemotherapeutic drugs, such as doxorubicin and paclitaxel, and protect them inside the nanoparticles [96]. In this way, the drug will not be recognized and excreted by transporter proteins until the nanoparticles are endocytosed by the cells and then enter into the tumor cells and release the drug. In addition, some nanoparticles can be degraded in the acidic or enzymatic environment inside the tumor cells, releasing a high concentration of the drug, further overcoming the effect of drug efflux [61].

The selective accumulation of nanoparticles at the tumor site can be enhanced by modifying some specific molecules on the surface of the nanocarriers, such as PEG or targeting ligands, which can increase the concentration of the drug in the tumor cells [97]. These modifications can also prolong the half-life of the drug in vivo and reduce its distribution in normal tissues. For example, by modifying folic acid on the surface of nanocarriers, tumor cells overexpressing folate receptors can be targeted, thus avoiding drug uptake by non-tumor cells and clearance by efflux proteins, and enhancing the efficacy of the drug at the tumor site [98].

### 4.2. Strategies to Reverse Tumor Drug Resistance

Reversing tumor drug resistance is a more aggressive therapeutic strategy aimed at restoring the sensitivity of cancer cells to drugs by directly interfering with or inhibiting the tumor drug resistance mechanism through specific nanocarrier design [99]. In recent years, researchers have developed a variety of nanosystems that have successfully reversed chemotherapeutic drug resistance [100]. Some nanocarriers have been designed to carry inhibitors of ABC transporter proteins, such as verapamil or other molecules, capable of inhibiting P-gp activity [101]. These carriers can be co-delivered with conventional chemotherapeutic drugs to release high concentrations of the chemotherapeutic drug while inhibiting drug efflux within the tumor cells [102]. For example, researchers have developed a polymeric nanoparticle capable of delivering both paclitaxel and an ABC transporter protein inhibitor, which significantly increased the accumulation of paclitaxel in MDR tumor cells and enhanced the chemosensitivity of the tumor [103].

A promising strategy to reverse tumor drug resistance is gene therapy [104]. By delivering specific gene editing tools (e.g., CRISPR-Cas9 system) or small interfering RNA (siRNA) to tumor cells, it is possible to down-regulate the expression or function of ABC transporter proteins, thus reversing the resistance mechanism of drug efflux [105]. Nanocarriers, particularly liposomes and polymer nanoparticles, have been shown to be effective in delivering these gene therapy tools to the tumor site and enabling precise genetic interventions [106]. For example, silencing the gene expression of P-gp using siRNA targeting has successfully reversed multidrug resistance in a variety of tumor models [107]. Nanocarriers provide an ideal platform for the co-delivery of multiple drugs or different types of therapeutic molecules [66]. These vectors can enhance the sensitivity of tumor cells to chemotherapy by co-delivering chemotherapeutic drugs with antioxidants, immunomodulators, or gene editing tools [108]. For example, it has been shown that nanocarriers co-delivering chemotherapeutic drugs with micro RNAs (miRNAs) or anti-apoptotic gene inhibitors can effectively disrupt tumor resistance mechanisms and significantly enhance the response of tumor cells to chemotherapeutic drugs [109].

### 4.3. Improvement of Intracellular Drug Release Efficiency

**Figure 6:**
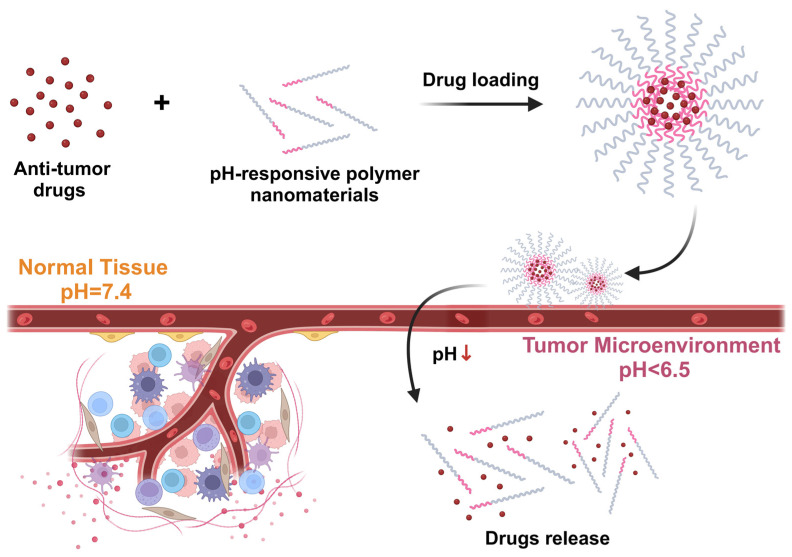
Mechanism of pH-responsive polymer nanomaterials for tumor-targeted drug delivery. Anti-tumor drugs are encapsulated into pH-responsive polymer nanomaterials through a drug-loading process. In normal tissues (pH = 7.4), the nanomaterials remain stable, minimizing premature drug release. Upon reaching the acidic tumor microenvironment (pH < 6.5), the pH-responsive polymers degrade, triggering the controlled release of the encapsulated drugs. This mechanism enhances drug delivery efficiency to tumor cells while reducing off-target effects in normal tissues. Created with BioRender.com.

The effective release of drugs inside tumor cells is the key to improving therapeutic efficacy, especially for drug-resistant tumors, and nanocarriers are able to ensure the effective release of drugs inside tumor cells through a variety of mechanisms [110,111]. Many tumor cells have lower pH values inside, especially in organelles such as lysosomes and endosomes [112]. Nanocarriers can rapidly release drugs upon entry into these low-pH organelles through pH-responsive mechanisms [34,113]. Figure 6 shows the mechanism of pH-responsive polymer nanomaterials for tumor-targeted drug delivery. For example, nanoparticles based on acid-sensitive polymers are able to rapidly depolymerize in the acidic environment inside the cell, releasing high concentrations of the drug and avoiding its premature release outside the cell [36]. This pH-responsive mechanism ensures the precise release of the drug inside the tumor cells while reducing the toxicity of the drug to normal tissues [114].

Another way to increase the efficiency of intracellular drug release is through photothermal or photodynamic effects [115]. Photothermal therapy utilizes near-infrared light irradiation to activate photothermal conversion materials in nanocarriers, such as gold nanoshells, graphene, or other photosensitive molecules, thereby converting light energy into heat energy, triggering local warming and promoting drug release from the carrier [116]. This process not only enhances the intracellular release of drugs, but also directly kills tumor cells through local heating. Photodynamic therapy, on the other hand, activates photosensitizers through light to produce ROS, which kill tumor cells and releases drugs [117]. Studies have shown that photodynamic nanocarriers can rapidly release chemotherapeutic drugs under light and synergize with ROS to improve tumor cell killing efficiency, especially in the treatment of drug-resistant tumors, which demonstrates significant effects [9].

### 4.4. Enhancement of the Immune System in Conjunction with Nanomedicine Delivery

In recent years, immunotherapy has made breakthroughs in the treatment of tumors, and nanomedicine delivery systems offer a unique opportunity to enhance immunotherapy [118,119]. Nanocarriers can carry immunomodulatory molecules or antigens delivered to the TME to stimulate the immune system to recognize and attack tumor cells [120,121]. By loading tumor antigens with adjuvants into nanoparticles, nanovaccines can increase the efficiency of antigen delivery and activate T-cell-specific immune responses against tumors [122]. For example, some studies have successfully activated a potent immune response against tumor cells using nanoparticles to deliver neoantigens [123]. Nanocarriers can also be co-delivered with immune checkpoint inhibitors, such as PD-1 or CTLA-4 inhibitors, to enhance the inhibition of tumor immune escape [119]. This co-delivery strategy significantly enhances the therapeutic effect of tumor immunotherapy. Nanotechnology offers several innovative strategies to overcome tumor drug resistance. By encapsulating drugs, inhibiting ABC transporter proteins, incorporating gene therapies, and improving the efficiency of drug release within the cell, nanodrug delivery systems provide effective solutions for reversing tumor drug resistance [95]. In addition, nanocarriers provide an ideal platform for combination therapy and immunotherapy [124]. With the continuous development and clinical validation of these technologies, nanodrug delivery systems will play an even more important role in future tumor therapy [57].

## 5. Biocompatibility and Safety of Nanomedicine Delivery Systems

During the development of nanodrug delivery systems, in addition to drug delivery efficiency and target specificity, biocompatibility and safety are key factors in evaluating their potential for clinical application [125]. Although nanotechnology offers many advantages for tumor therapy, its potential toxicity and adverse effects are still important barriers that limit its wide application [126,127]. Therefore, the toxicity, in vivo metabolic pathways, long-term safety, and immunogenicity of materials must be comprehensively considered when designing nanocarriers. The biocompatibility and safety of nanodrug delivery systems and their challenges and solutions during clinical translation will be discussed in detail below.

### 5.1. Toxicity of Nanocarriers

The toxicity of nanomaterials is one of the keys to the safety assessment of nanodrug delivery systems. The toxicity of nanocarriers may arise from their constituent materials, size, shape, surface properties, and behavior in vivo [128]. Certain nanomaterials may be inherently cytotoxic or trigger an immune response, leading to acute or chronic toxicity [129]. The following are a few common types of nanomaterials and their potential toxicity concerns.

Metal and metal oxide nanoparticles, such as gold nanoparticles, silver nanoparticles, and iron oxide nanoparticles, are widely used in oncology diagnosis and treatment due to their unique physicochemical properties (e.g., magnetic properties, photothermal effects) [130]. However, such nanoparticles may cause oxidative stress in vivo, inducing apoptosis or autophagy [131]. For example, iron oxide nanoparticles may damage cells by generating excessive ROS at certain concentrations [132]. Although surface modifications can reduce their toxicity, prolonged ingestion or high dose use may still lead to irreversible tissue damage. Carbon-based nanomaterials, such as carbon nanotubes and graphene, have become popular materials for oncology drug delivery and diagnosis due to their high mechanical strength and electrical conductivity [133]. However, carbon-based nanomaterials have high biopersistence and it is difficult to degrade or clear them in vivo, and their long-term use may trigger chronic inflammatory responses and organ toxicity [134]. Studies have shown that unmodified carbon nanotubes exhibit a high risk of pulmonary toxicity and fibrosis in animal models, thus requiring careful design and modification during application [135]. Polymer nanoparticles, such as PLGA and PEG-modified nanocarriers, are widely used for drug delivery due to their high biocompatibility and controlled degradation [70]. Most biodegradable polymers have a good safety profile, but the accumulation of degradation products and residual solvents may trigger local or systemic toxic reactions. For example, PLGA degradation produces lactic acid and glycolic acid, and although these metabolites are usually safely metabolized, they may still trigger local tissue acidosis at high doses [136].

### 5.2. Methods to Improve Biocompatibility

In order to reduce the toxicity of nanomaterials and improve their biocompatibility, researchers have developed several strategies to optimize the design of nanocarriers. The following are a few common approaches. By chemically modifying the surface of nanocarriers, their biocompatibility can be significantly improved and their toxicity reduced [137]. For example, PEG modification is a widely used technique to effectively reduce the interaction of nanoparticles with serum proteins by attaching PEG to the surface of the nanoparticles, reducing their risk of being recognized and cleared by macrophages, thus prolonging their circulation time and improving targeting [102]. In addition, some studies have shown that the tumor targeting of nanoparticles can be enhanced by modifying targeting ligands (e.g., antibodies, peptides, glycans), reducing nonspecific distribution to normal tissues and decreasing systemic toxicity [138]. For example, folic-acid-modified nanoparticles were able to target tumor cells expressing folate receptors, improving selective drug release and reducing toxicity to healthy tissues [109]. The use of biodegradable materials to construct nanocarriers can drastically reduce the in vivo retention time and its potential to induce long-term toxic reactions [139]. Polymer nanoparticles, such as PLGA and polylactic acid (PLA), are able to be degraded in vivo by hydrolysis or enzymatic degradation, releasing non-toxic or low-toxic metabolites [140]. By controlling the molecular weight and chemical structure of the polymers, the degradation rate of the nanoparticles can be regulated to achieve sustained drug release and reduce drug dose and toxic side effects [70]. Trigger-responsive nanocarriers are able to undergo depolymerization or drug release in specific in vivo environments (e.g., acid- or enzyme-reducing environments), thereby increasing the concentration of the drug at the tumor site and decreasing the impact on normal tissues [141]. For example, pH-responsive nanoparticles can rapidly release drugs under acidic conditions in the TME, reducing nonspecific release of drugs in normal tissues and improving therapeutic efficacy and safety [32].

### 5.3. Metabolism and Clearance of Nanodrug Delivery Systems

The metabolism and clearance pathways of nanocarriers in vivo are important aspects in their safety assessment [142]. An ideal nanodrug delivery system should have an effective metabolic pathway to ensure that it can be rapidly cleared after drug delivery to avoid long-term accumulation in the body [143]. Different types of nanocarriers have different metabolic pathways in the body [130]. Biodegradable polymer nanoparticles, such as PLGA and PLA, can be converted into small-molecule metabolites in vivo by enzymatic degradation and then excreted through the liver and kidney [70]. In contrast, it is often difficult for metal nanoparticles (e.g., gold and silver nanoparticles) and carbon-based nanomaterials to be biodegraded and there is more reliance on the macrophage system of the liver and spleen for clearance [144]. Metal nanoparticles may be excreted from the body through urine or feces, but long-term accumulation may still cause damage to organs [145]. Kidneys and livers are the main removal organs for nanocarriers [146]. In general, small-sized nanoparticles (less than 5–10 nm) are able to pass through glomerular filtration into the urine for excretion, while larger-sized nanoparticles (greater than 50 nm) are more likely to be taken up by the liver and pass through the biliary tract into the feces for excretion [147]. For some large-sized or non-degradable nanoparticles that are difficult to remove, they may accumulate in the liver, spleen, or lymph nodes for a long time, leading to chronic toxicity [130]. Therefore, the rational design of the size and surface properties of nanoparticles to optimize their clearance pathways is an important strategy to improve their safety [148].

### 5.4. Immune and Allergic Reactions

Immune and allergic reactions triggered by nanomedicine delivery systems are another safety issue to be considered [149]. Nanoparticles may be recognized as foreign substances by the immune system upon entry into the body, activating the complement system, macrophages, or dendritic cells and triggering a systemic immune response or local inflammation [150]. Certain nanoparticles, especially inorganic nanoparticles or carriers with cationic surfaces, may trigger strong immune responses or allergic reactions leading to acute or chronic inflammation [151]. By modifying “stealth” molecules (e.g., PEG) on the surface of nanocarriers, their probability of being recognized and cleared by the immune system can be reduced, thus prolonging the circulation time and reducing the immune response [152,153]. PEG modification technology has been widely used in a variety of nanomedicine delivery systems, which have demonstrated a good immune escape ability. In addition, several studies have explored the possibility of evading phagocytosis by macrophages by the surface modification of CD47 protein (“don’t eat me” signaling), thereby prolonging the half-life of nanoparticles in vivo [154,155,156].

In some cases, activating the immune system is, instead, an effective therapeutic strategy [120]. For example, by modifying tumor antigens or immune adjuvants on the surface of nanocarriers, the immune system can be stimulated to mount a specific immune response against tumor cells and enhance the anti-tumor effect [156]. Such nanovaccines have already achieved positive results in a variety of tumor immunotherapies [123]. The biocompatibility and safety of nanodrug delivery systems are crucial in their clinical applications [146]. The safety of nanocarriers can be effectively improved by surface modification, the use of biodegradable materials, and the rational design of metabolic and clearance pathways of nanoparticles. In addition, the modulation of the immune response and the evaluation of toxicity are critical steps to ensure the safe use of nanomedicines in the clinic [157,158]. In the future, with the further optimization of nanomaterials and more in-depth toxicological studies, nanodrug delivery systems will play a greater role in tumor therapy [159].

## 6. Challenges and Opportunities for Clinical Translation

The application of nanomedicine delivery systems in cancer therapy has achieved many laboratory-stage successes. However, translating these breakthroughs into clinical applications still faces many challenges and bottlenecks [160]. Although certain nanomedicines have entered clinical trials and even received approval from drug regulatory agencies, a series of technical, economic, and clinical issues still need to be resolved for large-scale dissemination and popularization [158]. In the following section, we will discuss in detail the challenges and opportunities faced by nanomedicines in the process of clinical translation and look ahead to their future development.

### 6.1. Current Status of Nanopharmaceuticals in Clinical Applications

With the advancement of nanotechnology, several nanomedicines have been approved for cancer treatment [161]. For example, liposome-based doxorubicin (Doxil), as the first FDA-approved nanomedicine, has been widely used in the treatment of several cancer types [162]. Doxil makes the drug more stable by encapsulating the doxorubicin and reduces the toxicity of conventional chemotherapeutic drugs [162]. In addition, paclitaxel nanoparticles (Abraxane) are another type of successful nanomedicine approved for the treatment of breast, non-small-cell lung, pancreatic, and ovarian cancers [163,164,165]. Abraxane uses albumin as a carrier, which dramatically improves the solubility and bioavailability of paclitaxel, and reduces severe allergic reactions [166]. These clinical applications show the great potential of nanodrug delivery systems, but they only represent the initial results of nanotechnology in tumor therapy. Nanomedicine delivery systems offer novel strategies for improving drug targeting, reducing side effects, and overcoming the limitations of the TME, and many novel nanomedicines are currently undergoing clinical trials, such as targeted radiotherapy based on gold nanoparticles and photothermal therapy based on carbon nanotubes [130,167,168]. Despite the promising future of nanomedicines, their widespread application still faces a series of challenges.

### 6.2. Translational Bottlenecks from the Laboratory to the Clinic

There are multiple challenges in promoting nanomedicines from the laboratory stage to clinical applications and achieving large-scale production. These challenges involve not only technical issues, but also regulatory, cost, and clinical validation aspects [158]. The production of nanomedicines requires highly accurate technical support to ensure consistency in size, shape, drug loading, and surface chemistry from batch to batch [169]. As the physicochemical properties of nanomaterials are highly sensitive at the molecular level, subtle process fluctuations may lead to significant performance differences, which is a major challenge during scaled-up production [170]. Therefore, maintaining consistency and stability in production is crucial to ensure safety and efficacy in clinical applications. However, the existing manufacturing technologies and equipment are still not fully adapted to the needs of large-scale nanomedicine production, and, in particular, there is still a huge gap in high-precision and high-efficiency manufacturing equipment [158].

In addition, the complexity of nanomedicines poses a great challenge to quality control [171]. It is difficult for existing assays to accurately determine the structure and functionalization of nanoparticles as well as their distribution and metabolic processes in the body [146]. To address these issues, the development of accurate quality control standards and advanced characterization techniques is an important task in clinical translation [172]. Although many nanomaterials have shown good biocompatibility in animal models, their long-term safety still needs further validation [146]. Certain nanoparticles may accumulate in the body, especially non-degradable or difficult-to-biodegrade nanomaterials, such as metal nanoparticles and carbon nanotubes, which may trigger long-term inflammatory responses or other chronic diseases [135]. In addition, the interaction of nanoparticles with the immune system is not well understood [173]. Some nanomedicines may trigger unexpected immune responses, such as complement activation and acute allergic reactions; thus, in-depth toxicological studies and long-term effects assessment are crucial in clinical trials. The highly complex and unpredictable in vivo behavior of nanomedicines is also a major obstacle in their clinical translation [130]. The distribution, metabolism, and clearance of nanoparticles in vivo are influenced by a variety of factors, including particle size, shape, surface charge, and functionalization modifications [172]. Physiological differences in different patients (e.g., vascular permeability, differences in TME, metabolic rate) also affect the in vivo behavior of nanomedicines, leading to inconsistent therapeutic effects [174]. Therefore, designing nanocarriers with highly controllable and stable in vivo behaviors becomes the key to improve clinical success [146].

Due to the complexity of the nanodrug delivery system, its approval process is also more complicated than that of traditional drugs [175]. The current review standards for nanomedicines by major drug regulatory agencies around the world are not yet perfect, especially for the long-term toxicity, metabolic pathways, and immuno-compatibility of nanomaterials, which lacks unified regulatory guidance. This leads to a lengthy approval process for novel nanomedicines, further increasing development costs and market entry [158,175]. In order to facilitate the clinical translation of nanomedicines, there is an urgent need to establish a comprehensive regulatory system and develop a standardized review process that meets the characteristics of nanomedicines.

### 6.3. Future Clinical Prospects for Nanopharmaceuticals

Despite the challenges, nanomedicine delivery systems also hold great opportunities in clinical translation [161]. The development of nanotechnology has brought a new perspective to personalized medicine and precision medicine, especially in the field of oncology, where nanomedicines can be delivered in a targeted manner to achieve highly specific treatment with low side effects [130]. Nanomedicine delivery systems can specifically target molecular markers of tumor cells through surface modification for precise drug delivery and reduce systemic toxicity [137]. With the development of gene sequencing and molecular diagnostic technology, personalized nanodrug delivery systems can be designed in the future for precision therapy based on patient-specific genotypes, tumor types, and microenvironmental characteristics [93]. This can not only improve drug efficacy, but also reduce the incidence of drug resistance and recurrence. For example, the delivery of siRNA or CRISPR-Cas9 gene editing tools via nanocarriers to inhibit or edit specific genes associated with tumors will open up new directions for personalized therapy [175].

The versatility of nanocarriers makes them an ideal platform for combination therapy [165]. In cancer treatment, the combined application of various strategies such as chemotherapy, gene therapy, immunotherapy, and radiotherapy has shown better therapeutic effects [146,176]. Nanodrug delivery systems can enhance the synergistic effect of therapy through multi-drug co-loading and combined delivery of anti-tumor drugs and immunomodulatory molecules [44]. For example, nanocarriers can simultaneously deliver chemotherapeutic drugs and immune checkpoint inhibitors (e.g., PD-1 inhibitors) to enhance the tumor immune response and inhibit the immune escape mechanism of tumors, which, in turn, improves the antitumor efficacy [50].

Nanodrug delivery systems can also be combined with other cutting-edge technologies to further improve therapeutic efficacy [93]. For example, AI technology is used to optimize the design of nanomedicine delivery, predict the behavior and efficacy of nanoparticles in vivo, and shorten the drug development cycle [177,178]. Another example is the combination of nanomedicines with smart medical devices (e.g., wearable sensors, in vivo microdevices) to realize the real-time monitoring and dynamic adjustment of therapeutic regimens, thus improving the precision and safety of treatment [179]. Despite the remarkable progress of nanomedicine delivery systems in the laboratory stage, their clinical translation still faces many challenges such as mass production, quality control, safety assessment, and regulatory oversight [158]. However, with the continuous progress of technology and the gradual improvement of regulatory policies, the application of nanodrug delivery systems in cancer therapy is promising. In the future, nanomedicines are expected to further improve the efficacy of oncology treatment and promote the realization of precision medicine by combining personalized treatment, combination therapy, and emerging technologies [93].

## 7. Summary and Outlook

Nanotechnology has made significant advancements in the field of drug delivery systems in recent decades, particularly in the targeting of TMEs and the overcoming of tumor drug resistance. The precise design of nanomedicine drug delivery systems enables them to target key characteristics of the TME, such as hypoxia, acidic conditions, and high interstitial pressure. This improves the distribution of drugs in tumor tissue and enhances treatment effects. Furthermore, by encapsulating chemotherapeutic drugs, gene therapy tools, or immunomodulatory molecules, nanocarriers can not only bypass the multidrug resistance mechanism of tumors, but also reverse drug resistance and enhance drug efficacy.

Despite numerous research breakthroughs, nanomedicine still encounters significant challenges in the context of clinical translation. Key issues that impede the widespread application of nanomedicine include inconsistency in mass production, uncertainty in drug metabolism pathways, the assessment of long-term safety, and limitations of the current regulatory system. However, with the continuous advancement of technology and the gradual improvement of regulatory policies, nanotechnology is poised to play a more pivotal role in the future treatment of cancer.

As molecular biology and genomics advance, nanomedicine will increasingly focus on the design of personalized treatment plans. By targeting tumor molecular markers specific to the patient, nanomedicine can achieve higher specificity and better treatment effects. Furthermore, the combination of nanotechnology with new treatment methods such as gene therapy and immunotherapy offer promising new avenues for overcoming tumor drug resistance.

The future development of nanomedicine will depend on the integration of multidisciplinary technologies. The combination of nanotechnology with emerging technologies such as AI and big data analysis enables the accurate modeling and prediction of the in vivo behavior of nanomedicines, thereby optimizing vector design. Furthermore, the integration of smart medical devices (such as in vivo sensors and wearable devices) with nanomedicines facilitates the real-time monitoring and dynamic adjustment of treatment plans, thus enhancing the precision and safety of treatment.

## 8. Conclusions

Nanodrug delivery systems have revolutionized tumor therapy like never before. By overcoming the complexity of the TME and bypassing drug resistance mechanisms, nanotechnology has shown great potential to improve drug efficacy and reduce toxic side effects. Despite the challenges of clinical translation and long-term safety, the application of nanomedicines in cancer therapy has an unlimited future as technology advances and emerging sciences converge. In the near future, nanotechnology will surely inject a strong impetus to the development of personalized medicine and precision medicine, and provide a new hope for mankind to overcome cancer.

## Data Availability

No new data were created or analyzed in this study. Data sharing is not applicable to this article.
